# Effects of UV-Ozone Treatment on Sensing Behaviours of EGFETs with Al_2_O_3_ Sensing Film

**DOI:** 10.3390/ma10121432

**Published:** 2017-12-15

**Authors:** Cuiling Sun, Ruixue Zeng, Junkai Zhang, Zhi-Jun Qiu, Dongping Wu

**Affiliations:** 1 State Key Laboratory of ASIC and System, Fudan University, Shanghai 200433, China; 15210720082@fudan.edu.cn (C.S.); 13110720024@fudan.edu.cn (R.Z.); 15110720077@fudan.edu.cn (J.Z.); 2 School of Information Science and Technology, Fudan University, Shanghai 200433, China

**Keywords:** EGFETs, Al_2_O_3_, pH sensing, instability, UV-ozone

## Abstract

The effects of UV-ozone (UVO) treatment on the sensing behaviours of extended-gate field-effect transistors (EGFETs) that use Al_2_O_3_ as the sensing film have been investigated. The Al_2_O_3_ sensing films are UVO-treated with various duration times and the corresponding EGFET sensing behaviours, such as sensitivity, hysteresis, and long-term stability, are electrically evaluated under various measurement conditions. Physical analysis is also performed to characterize the surface conditions of the UVO-treated sensing films using X-ray photoelectron spectroscopy and atomic force microscopy. It is found that UVO treatment effectively reduces the buried sites in the Al_2_O_3_ sensing film and subsequently results in reduced hysteresis and improved long-term stability of EGFET. Meanwhile, the observed slightly smoother Al_2_O_3_ film surface post UVO treatment corresponds to decreased surface sites and slightly reduced pH sensitivity of the Al_2_O_3_ film. The sensitivity degradation is found to be monotonically correlated with the UVO treatment time. A treatment time of 10 min is found to yield an excellent performance trade-off: clearly improved long-term stability and reduced hysteresis at the cost of negligible sensitivity reduction. These results suggest that UVO treatment is a simple and facile method to improve the overall sensing performance of the EGFETs with an Al_2_O_3_ sensing film.

## 1. Introduction

The ion-sensitive field-effect transistor (ISFET) was first developed by Bergveld in 1970, which were initially used for pH detection and ion concentration measurement [1,2]. ISFETs are potentiometric devices based on the structure of the metal-oxide-semiconductor field-effect transistor (MOSFET) by removing the metal gate of the MOSFET [1,2,3]. When an ISFET is used for pH sensing, the electric potential at the insulator/electrolyte interface will be controlled by the hydrogen ion concentration with the bare oxide contacting the chemical environment [3,4]. As a modified category of the ISFET, extended-gate field-effect transistor (EGFET) based on the same working principle of the ISFET is comprised of a sensing structure and a conventional MOSFET [5,6]. The sensing structure with a sensing film contacting the electrolyte directly is connected to the FET metal gate by a metal signal line. Without immersing the FET in the electrolyte and removing the metal gate, the EGFET presents better thermal stability and light stability compared to the ISFET [6,7,8,9]. The EGFET allows the reuse of the MOSFET and a flexible shape of the sensing structure. Over these years, materials used as EGFET sensing films have been investigated, such as tin dioxide (SnO_2_), tantalum pentoxide (Ta_2_O_5_), vanadium pentoxide (V_2_O_5_), and so on [7,9,10]. Among these materials, Al_2_O_3_ is considered as a suitable sensing material from the point of view of its inert characteristic, high sensitivity, good ion selectivity, and compatibility with the complementary metal oxide semiconductor (CMOS) process [11]. With the advantages of light insensitivity, easy fabrication, low cost, and disposable gate, EGFETs have drawn a great deal of attention and a wide range of applications based on EGFETs have been reported [5,6,7,8,9,12,13,14,15]. However, several instability phenomena related to the sensing film still exist which limits the measurement precision when an EGFET is used for pH sensing [16,17]. According to previous studies [17,18,19,20,21,22], the instability is mainly caused by defects in the sensing film, such as buried sites, charge traps, and slow-reacting surface sites. For example, hysteresis can be attributed to buried sites near the sensing film surface [22]. Hysteresis leads to a response deviation between identical pH measurement conditions, causing an unreliable measurement output [17,23]. Therefore, it is necessary to utilize a high-quality film with few defects as the sensing film [24]. It is generally accepted that the sensing mechanism can be explained with site dissociation model, meaning that the reaction between hydrogen in the electrolyte and amphoteric groups takes place at the surface of the sensing film [4,25,26]. The quality of the sensing film surface demonstrably plays a significant role in EGFET sensing behaviours and is sensitive to surface contamination, surface morphology, and defects. To improve the quality of sensing film, several surface modification methods have been attempted, including oxygen plasma, SF_6_ plasma, wet chemical treatment, and the texturization of the sensing film [24,27,28,29,30,31]. Among these surface treatment methods, UV-ozone (UVO) treatment is a typical method for surface cleaning or surface activation during sensor fabrication [32,33,34,35,36,37,38,39,40,41]. UVO treatment is reportedly capable of removing organic contaminants and has the advantages of being a dry cleaning agent, easy to use, harmless to the environment, and compatible with the CMOS process [42,43].

In order to investigate the comprehensive effects of UVO treatment in EGFET sensing behaviours, this study conducted a series of experiments with various UVO treatment times on Al_2_O_3_ sensing film. With the introduction of the UVO treatment, a large enhancement on sensing performance was observed, especially in the reduction of hysteresis. In order to clarify the mechanism of UVO treatment, X-ray photoelectron spectroscopy (XPS) and atomic force microscopy (AFM) were utilized to analyse surface composition and morphology. XPS and AFM results confirmed that a cleaner and smoother Al_2_O_3_ sensing film surface was obtained after UVO treatment. Moreover, the –OH groups involved in the Al_2_O_3_ deposition process had been removed. Resultantly, an optimal UVO treatment time was, therefore, obtained.

## 2. Materials and Methods

### 2.1. Fabrication Process

**Figure 1:**
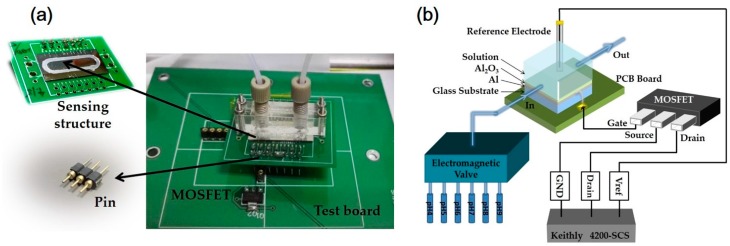
(**a**) Image of sensing structure connected to a commercial MOSFET on a test board; and (**b**) a schematic diagram and the measurement setup of EGFETs.

The following is the process by which EGFET fabrication based on Al_2_O_3_ sensing film was achieved. The EGFET is separated into two parts: one is a sensing structure with an extended gate and the other is a commercial MOSFET. The sensing structure has a glass substrate, and a metal electrode is deposited on the substrate, which is connected to the metal gate of the MOSFET. When the sensing film contacts with the electrolyte, electric potential at the sensing film surface will vary with the ion concentration. Therefore, the electric potential variation at electrolyte/insulator interface will be detected by the MOSFET. To fabricate the sensing structure, the glass substrate (CORNING EAGLE XG^®^, CORNING, New York, NY, USA) was cleaned with standard RCA cleaning process, and 50 nm titanium (Ti) and 500 nm aluminium (Al) were deposited on the substrate using electron beam evaporation as an electrode. Next, 50 nm Al_2_O_3_ films were deposited by atomic layer deposition (ALD) on the Ti/Al electrode under 300 °C. The thickness of Al_2_O_3_ film was analysed by an ellipsometer. The dimension of Al_2_O_3_ films was 2 cm in length and 1.2 cm in width. Then, the sensing structures were treated with a UVO cleaner (BZZ250G-T, HWOTECH, Shenzhen, China). The UV intensity was 25 mW/cm^2^ at 254 nm, and ozone was generated in air atmosphere by absorbing 185 nm UV radiation. After UVO treatment, the sensing structures were pasted on the copper line of a printed circuit board (PCB) with adhesive tape. After bonding with the metal pad of the PCB, an epoxy resin adhesive of 200 nm thickness was coated on the sensing film surface following an elliptical path using a dispensing robot (TH-2004D-K, Tianhao, Zhejiang, China), which served as a container for the flow of liquid, as well as for sealing. When the epoxy was dry, a poly(methyl methacrylate) (PMMA) board was placed on it and fixed with screws on the four corners of the PCB. Finally, as shown in Figure 1a, this PCB was connected to a commercial n-channel power MOSFET gate (MMFT960T1, ON Semiconductor Corporation, Phoenix, AZ, USA) on a test board using pins.

### 2.2. Electrical and Physical Measurements

The device diagram and electrical measurement setup are demonstrated in Figure 1b. A stable environment for pH measurement was maintained by controlling the flow channel microfluidically. The Ag/AgCl electrode was inserted in the pH buffer solution for use as a reference electrode. Next, standard pH buffer solutions (Reagecon, Shannon, Ireland) with pH values varying from 4 to 9 were used for pH measurement. The electrolyte/insulator interface that all the samples were immersed in was stabilized in the buffer solution (pH 7) for at least 24 h before pH measurement. The electrochemical characteristics of the ALD Al_2_O_3_ EGFETs were obtained using a Keithley 4200-SCS semiconductor parameter analyser (Keithley, Cleveland, OH, USA). In the steady-state measurement, a reference voltage (*V*_ref_) applied at the Ag/AgCl reference electrode was swept from −1 V to 5 V with a step size of 0.01 V and then backwards, while a constant source-drain voltage was kept at 0.1 V. In the real-time measurement condition, the device was operated in the linear region by setting the reference voltage *V*_ref_ at 3.5 V. As the pH in the loop changed from pH 9 to pH 4 and back, the source-drain current (*I_ds_*) was measured. Each pH value was held constant for 60 s before being altered and this measurement was controlled by an electromagnetic valve. The sensing behaviours of the devices were monitored over a period of three weeks. Using this methodology, the sensing behaviours of sensitivity, hysteresis, and stability were obtained. To acquire comprehensive knowledge of the material surface condition, XPS analysis and AFM analyses were also performed. The surface content was analysed by a Kratos AXIS Ultra DLD XPS (Kratos, Kyoto, Japan) using an Al K*α* X-ray source (1486.6 eV). The surface morphology was determined using a Bruker Dimension Icon AFM (Bruker, Karlsruhe, Germany) over a scan area of 5 × 5 μm^2^ in tapping mode under ambient conditions. The AFM images were taken in the centre of the sensing film for each sample.

## 3. Results and Discussion

### 3.1. EGFET Sensing Behaviours

**Figure 2:**
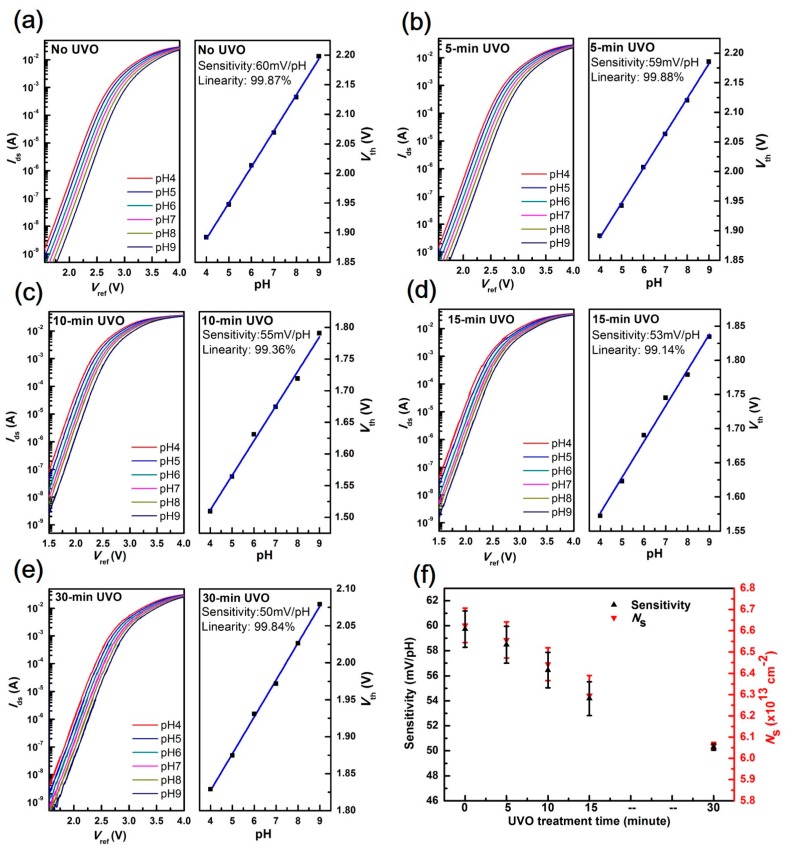
Transfer characteristics, sensitivity and linearity of EGFETs using 50 nm-Al_2_O_3_: (**a**) without UVO treatment; and with UVO treatment for (**b**) 5; (**c**) 10; (**d**) 15; and (**e**) 30 min; and (**f**) a comparison of average sensitivity and *N_s_* of three EGFETs (*N* = 3) without UVO treatment and with UVO treatment for 5, 10, 15, and 30 min.

Figure 2 illustrates the transfer and sensitivity characteristics of EGFETs over different UVO treatment times. As shown in the left diagram of Figure 2a–e, the transfer curves shifted positively as the pH increased, reflecting the surface potential of the sensing film increasing with hydrogen ion concentration. The threshold voltage *V*_th_ of the EGFETs was defined as *V*_ref_ when the drain current reached 1 × 10^−7^ A, and sensitivity was obtained by linearly fitting *V*_th_ with different pH values.

According to past studies [44,45,46], the sensitivity can be expressed as:

(1) $$\mathrm{sensitivity}=2.3\frac{kT}{q}\alpha ,\;\mathrm{with}\;\alpha =\frac{1}{1+\frac{2.3kTC_{di}}{q^{2}\beta_{int}}}$$

where *k* is the Boltzmann’s constant, *T* is the absolute temperature, *q* is the elementary charge, *β_int_* is the intrinsic buffer capacity, and *C_di_* is the differential capacitance. The experiment was carried out at room temperature. Sensitivity is influenced by two key parameters: buffer capacity *β_int_* and differential capacitance *C_di_* [45]. *β_int_* characterizes the ability of the surface to buffer small pH changes at the sensing film surface, which depends solely on the intrinsic properties of the sensing material, i.e., the equilibrium constants of *K_a_* and *K_b_* and the surface site number *N_s_* [25,45]. *β_int_* can be expressed as:

(2) βint=2q2Ns (KaKb)12kTCdi

As for Al_2_O_3_, the related parameters were set as *K_a_* = 10^−10^ and *K_b_* = 10^−6^ [46]. *C*_dif_ is defined as:

(3) $$C_{\mathrm{dif}}=\frac{q\sqrt{8\varepsilon_{w}kTc}}{2kT}$$

where *c* is the electrolyte concentration (*c* = 0.1 mol^−1^) and *ε_w_* is the dielectric electrolyte constant (*ε_w_* = 78.5).

Therefore, *C_di_* can be set to the constant value *C_di_* = 16 μF/cm^2^ as discussed in [25,44,45]. When surface amphoteric groups react with hydrogen ions, the surface sites exist in three forms: Al-O^−^, Al-OH_2_^+^ and Al-OH [44,46]. Therefore, surface site number *N_s_* is the sum of these three sites [44,46]. Based on Equations (1)–(3), *N_s_* can be calculated from the measured sensitivity in our experiment. Figure 2f shows that a reduced *N_s_* caused by UVO treatment results in a decreased sensitivity. The *N_s_* of 30-min UVO-treated Al_2_O_3_ film is 6.06 × 10^13^ cm^−2^, which is 8% smaller than that of the as-fabricated one. And the 30-min UVO-treated sample has the smallest sensitivity of 50 mV/pH, which is decreased by 17% compared with the untreated one. Therefore, *N_s_* as well as sensitivity, shows an obvious degradation with long time exposure to UVO. With short time exposure to UVO, the reduction of *N_s_* is smaller than 3% and sensitivity presents a small degradation. It can be seen in Figure 2f that *N_s_* shows a monotonic decay with increased treatment time, and sensitivity is strongly correlated with *N_s_*.

**Figure 3:**
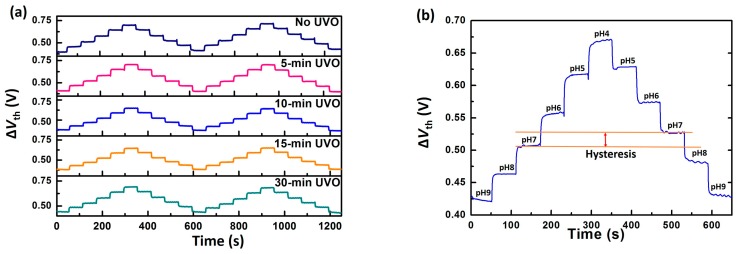
(**a**) Real-time measurement results for EGFETs with different UVO treatments; and (**b**) real-time curve to evaluate hysteresis width at pH 7 over pH loops in the sequence pH 9–4 and pH 4–9.

To acquire further knowledge of EGFET sensing behaviours, a real-time measurement was performed using a stepwise pH progression from pH 9 to pH 4, and back, while keeping *V*_ref_ constant. It is shown in Figure 3a that all the pH loops in the real-time curve for each device are reproducible. The shift of threshold voltage Δ*V*_th_ was calculated using the transconductance *g_m_*, $\Delta V_{\mathrm{th}}=\frac{\Delta I_{ds}}{g_{m}}$. As demonstrated in Figure 3b, Δ*V*_th_ does not coincided with the same pH in the sensing loop, leading to a so-called hysteresis phenomenon. Here, the hysteresis width is defined as the deviation between Δ*V*_th_ from the neighbouring pH 7.

Table 1 shows the hysteresis width varying with UVO treatment time. All devices with the UVO treatment can be seen to have a smaller hysteresis as compared to the as-fabricated one. The smallest hysteresis was obtained in the 10-min UVO-treated device, which reached 10 mV, about 66% smaller than that of the untreated one. When the treatment time lasts for over 10 min, the hysteresis increases again and approaches the value of the untreated device. UVO treatment can, therefore, be used to effectively decrease EGFET hysteresis with an optimal time of 10 min. In generally, the EGFET response to pH variation happens in milliseconds, which is characterized by fast response [19,22,47]. However, there is an extra time needed for a saturated response, where slow response or hysteresis takes place and causes a response delay in the order of minutes to hours after pH variation [22]. It is commonly accepted that a fast response is dominated by the surface effect, where surface amphoteric groups react with hydrogen ions in the electrolyte. As for hysteresis, it is attributed to defects in the sensing film, which have a slow response to pH variation. These defects can be explained in terms of the interior OH sites in the sensing film, which are created during the fabrication process or exposure to the electrolyte [22]. Since those sites are located beneath the sensing film surface, it takes a longer time for the bulk response between buried sites and hydration ions [22,48]. With regard to the Al_2_O_3_ sensing film deposited by ALD, it is well known that Al-OH bonds are formed during reaction between trimethylaluminum (TMA) and H_2_O [49,50,51]. It is reasonable to ascribe the presence of interior OH sites to Al-OH caused by deposition. These buried OH sites near the sensing film surface can also respond to pH variation, which incurs hysteresis. It can be inferred that the O-H bond will be broken under the radiation of UV light, which results in Al-OH reduction [50,52,53,54].

**Figure 4:**
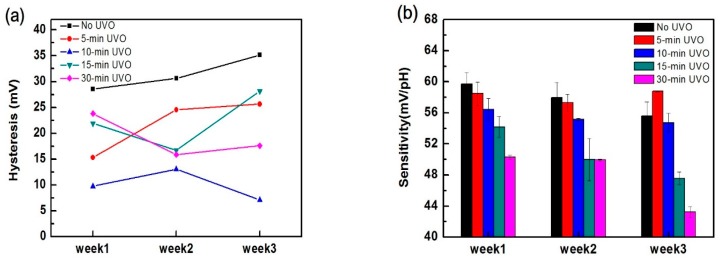
(**a**) Hysteresis characteristics and (**b**) sensitivity characteristics of EGFETs using 50 nm-Al_2_O_3_ without and with UVO treatment over three weeks. The lines in (**a**) are guides to the eye.

To investigate the long-term stability of UVO-treated devices, sensing behaviours were evaluated continuously over three weeks. The comparisons of sensitivity and hysteresis characteristics during three weeks’ time are shown in Figure 4. The performance of untreated devices degraded with time and their sensitivity was reduced to 56 mV/pH. The sensing performance of 10-min UVO-treated devices was consistent over three weeks’ time, showing no remarkable degradation in sensitivity and hysteresis. By contrast, 5-min, 15-min, and 30-min UVO-treated devices were not equipped with good long-term stability and their sensitivity decreased or hysteresis increased within the three week period. As a result, the device with UVO treatment for 10 min provides a stable overall sensing performance over a long period of time. With extended exposure to UVO, the sensing film may be covered by a corrosive mist in the presence of water vapour or nitrogen and sulphur oxides in the air [52,53,54]. Therefore, the device shows a certain degree of degeneration when treatment time exceeds 10 min.

### 3.2. Sensing Film Surface Analysis

**Figure 5:**
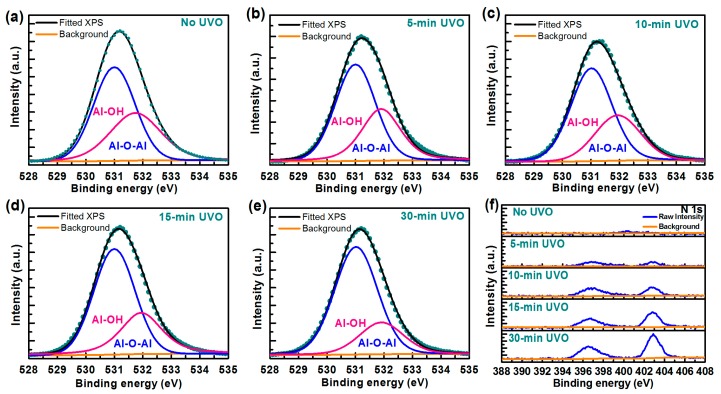
XPS O 1s core level spectra of Al_2_O_3_: (**a**) without UVO treatment; and with UVO treatment for (**b**) 5; (**c**) 10; (**d**) 15; and (**e**) 30 min; and (**f**) XPS N 1s core level spectra of Al_2_O_3_ with different UVO treatment.

To clarify the mechanism underlying the UVO treatment, surface analysis was conducted using XPS and AFM. XPS was used to investigate the chemical composition of Al_2_O_3_ films with different treatment times. The O 1s core level spectra are shown in Figure 5a–e. According to the Gaussian fitting, the O 1s peak is de-convoluted into two components: one at 531.9 ± 0.1 eV, corresponding to Al-OH bonds, and the other at 531.0 eV, corresponding to Al-O-Al bonds. Table 2 provides detailed composition information for the Al_2_O_3_ films. The area ratio of Al-O-Al to Al-OH became higher and the surface carbon element became lower as UVO treatment time increased. As mentioned above, the Al-OH bonds were introduced into the Al_2_O_3_ films when TMA reacted with H_2_O during the ALD process [50,51]. The –OH groups near the surface acted as buried sites resulting in hysteresis, so hysteresis was reduced with –OH groups decreasing after the UVO treatment [22,50]. However, hysteresis showed an increased tendency when treatment time exceeded 10 min. The non-monotonic behaviour of hysteresis can be explained in terms of nitride formation with prolonged treatment time, as shown in Figure 5f. The increase of nitride species resulted in hysteresis increasing [52,53,54]. The control of treatment time can help minimize the influence of nitride species and benefit from reduced Al-OH content. Therefore, an optimal treatment time of 10 min yielded the smallest hysteresis. Moreover, a cleaner surface was obtained when the surface carbon-related contaminates were removed by UVO. According to the XPS results, UVO treatment for an appropriate time can improve Al_2_O_3_ film quality as well as EGFET sensing performance by reducing –OH group content and surface contaminates.

**Figure 6:**
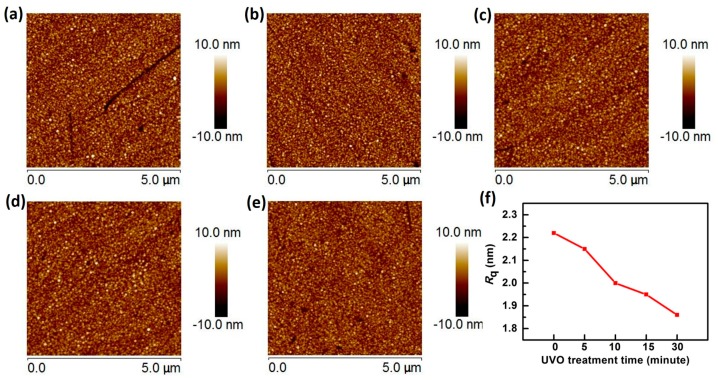
AFM images of Al_2_O_3_ (**a**) without UVO treatment and with UVO treatment for (**b**) 5; (**c**) 10; (**d**) 15; and (**e**) 30 min; and (**f**) *R*_q_ as a function of UVO treatment.

To obtain full knowledge of UVO effects on sensitivity, AFM analysis is conducted. The sensing film surface morphology is shown in Figure 6a–e and surface roughness is characterized by *R*_q_ (root mean square) is considered as a function of UVO treatment time in Figure 6f. The change in *N_s_* can be characterized by *R*_q_ from AFM image [28,55]. It is shown in Figure 6 that surface roughness gets smaller and the surface gets smoother with UVO treatment. The smallest *R*_q_ is observed in the 30-min UVO-treated samples, which was decreased by 16% compared with 2.22 nm of the untreated samples. Surface roughness of the Al_2_O_3_ samples decreased as a function of UVO treatment time, reflecting that *N_s_* was reduced with UVO treatment, which caused the sensitivity degradation as discussed above. AFM results indicate that *N_s_* is decreased with increased treatment time, which is consistent with Figure 2f.

## 4. Conclusions

The effects of UVO treatment on the sensing behaviours of EGFETs based on Al_2_O_3_ sensing film have been studied. With the introduction of UVO treatment, EGFETs exhibit excellent pH sensing characteristics, such as decreased hysteresis, good long-term stability, and high sensitivity. Though the sensitivity decreases with increasing treatment time, the lowest sensitivity is still 50 mV/pH under UVO treatment for 30 min. The AFM and XPS results reveal that smoother sensing film surface, as well as reduced carbon-related contaminations and a decreased number of buried sites in the sensing film are achieved after UVO treatment. As a result, UVO treatment is effective to provide a higher-quality sensing film with a cleaner and smooth surface, as well as fewer buried sites. Furthermore, the 10-min UVO-treated device exhibits smallest hysteresis, best long-term stability, and yet still high enough sensitivity. Hence, UVO treatment is a very promising technique to improve the quality of the sensing films, as well as overall sensing performance of the corresponding EGFETs.

## Figures and Tables

**Table 1:** Hysteresis characteristics of EGFETs using 50 nm-Al_2_O_3_ without UVO treatment and with UVO treatment for 5, 10, 15, and 30 min.

Treatment	No UVO	5-min UVO	10-min UVO	15-min UVO	30-min UVO
Hysteresis (mV)	28	15	10	22	24

**Table 2:** Elemental composition in the surface and bulk based on XPS measurement. Values are given in at %.

Treatment	Surface	Bulk		
	C (Atom %)	Al-O-Al (Area %)	Al-OH (Area %)	Al-O-Al/Al-OH (Area Radio)
No UVO	12.59	59.35	40.65	1.46
5-min UVO	12.21	62.76	37.24	1.69
10-min UVO	9.56	65.62	34.38	1.91
15-min UVO	9.45	66.91	33.09	2.02
30-min UVO	9.27	75.72	24.28	3.12
